# Assessment of Steatosis and Fibrosis in Liver Transplant Recipients Using Controlled Attenuation Parameter and Liver Stiffness Measurements

**DOI:** 10.1155/2021/6657047

**Published:** 2021-02-08

**Authors:** Ivana Mikolasevic, Goran Hauser, Maja Mijic, Viktor Domislovic, Delfa Radic-Kristo, Zeljko Krznaric, Melanija Razov-Radas, Tajana Pavic, Marija Matasin, Tajana Filipec Kanizaj

**Affiliations:** ^1^Department of Gastroenterology, University Hospital Center Rijeka, Rijeka, Croatia; ^2^Department of Gastroenterology, University Hospital Merkur, Zagreb, Croatia; ^3^Faculty of Medicine, Rijeka, Croatia; ^4^Faculty of Health Studies, Rijeka, Croatia; ^5^Department for Gastroenterology and Hepatology, University Hospital Center Zagreb, Zagreb, Croatia; ^6^Department of Hematology, University Hospital Merkur, Zagreb, Croatia; ^7^Faculty of Medicine, Zagreb, Croatia; ^8^Division of Gastroenterology, Department of Internal Medicine, Zadar General Hospital, Zadar, Croatia; ^9^Department of Internal Medicine, Division of Gastroenterology and Hepatology, University Hospital Center “Sestre Milosrdnice”, Zagreb, Croatia

## Abstract

**Aim:**

The primary objective of this study was to evaluate the prevalence of increased controlled attenuation parameter (CAP) and liver stiffness measurements (LSM) as surrogate markers of liver steatosis and fibrosis in liver transplant recipient (LTR). Secondary objectives were to determine the predictors of increased CAP and LSM in population of LTR.

**Methods:**

In this prospective, cross-sectional study, we have evaluated 175 LTRs' mean age as 61 (53–65) with a functioning graft for more than one year who came for regular outpatient examinations to the Department of Gastroenterology, University Hospital (UH) Merkur, Zagreb, Croatia.

**Results:**

Of 175 analyzed LTRs, 34.28% had obesity, 64.00% had hypertension, 38.28% had diabetes, and 58.85% had hyperlipidemia. The prevalence of liver steatosis was 68.57%, while the prevalence of severe liver steatosis was 46.85%. On multivariate analysis, independent factors associated with liver steatosis were male gender, total cholesterol as positive predictor, and HDL as negative predictor, and independent factors positively associated with severe liver steatosis were higher body mass index (BMI) and higher triglyceride levels. The prevalence of moderate liver fibrosis was 54.85%, while the prevalence of advanced liver fibrosis was 24%. On multivariate analysis, independent factors positively associated with moderate fibrosis were gamma-glutamyl transferase (GGT) and CAP, while the independent factor positively associated with advanced fibrosis was GGT.

**Conclusion:**

Our study showed high prevalence of increased CAP and LSM measurements as surrogate markers of liver steatosis and fibrosis. Metabolic syndrome components were highly present and were associated with CAP and LSM values as well as in the pretransplant setting. Due to high prevalence of metabolic comorbidities and nonalcoholic fatty liver disease in LTRs and the lack of the abnormal liver test in a significant number of these patients, TE with CAP may be a reasonable initial assessment for LTRs with one or more components of the metabolic syndrome.

## 1. Introduction

The prevalence of obesity, diabetes mellitus type 2, and metabolic syndrome (MetS) is increasing; therefore, nonalcoholic fatty liver disease (NAFLD) is becoming the most important chronic liver disease (CLD) today. According to the data, NAFLD affects around 25% of the total population. NAFLD is a liver manifestation of MetS and is in close relationship with MetS and its individual components (i.e., diabetes mellitus type 2, obesity, dyslipidemia, and hypertension). Today, we evaluate NAFLD as a multisystem disease because in the past ten years, a large amount of data had connected NAFLD with numerous extrahepatic chronic diseases such as cardiovascular diseases (CVD), chronic kidney disease (CKD), and type 2 diabetes mellitus. [1]. A subset of NAFLD patients will develop end-stage liver disease (ESLD) (i.e., cirrhosis and/or hepatocellular carcinoma (HCC)) [1–7]. Additionally, emerging results suggest that HCC can evolve even in noncirrhotic NAFLD [3]. Fatty liver disease is of great interest for many authors who manage patients with liver transplant because it has multiple impacts in the context of liver transplantation (LT) [3, 8]. For the first, NAFLD-related ESLD (i.e., cirrhosis and HCC) has become one of the leading indications for LT in the USA. It is expected that NAFLD will become the leading indication for LT in the next 20 years due to epidemic raise in the incidence of MetS and its individual components [3, 8, 9]. Second, the challenging issue in the context of NAFLD and LT is also a liver allograft steatosis which is in direct relationship with a pool of potential donors. Because of epidemic raise of MetS (and consequently NAFLD) in the next decade, we can expect more donors with fatty liver disease [3, 8, 9], and consequently, a great proportion of potential organ donors will be rejected for LT use [3, 8]. Third, NAFLD patients often have multiple comorbidities, thus making LT a challenging procedure for them. Finally, in the post-LT setting, there are several challenging issues for NAFLD such as de novo NAFLD or recurrent NAFLD, as well as the risk for CKD and CVD [3].

With the help of LT, survival of patients with liver failure (acute or secondarily to cirrhosis) as well as those with HCC has significantly improved. Due to the progress in transplant surgery and in modern immunosuppressive therapy, early post-LT morbidity and mortality has decreased. Consequently, the focus of transplant doctors is changing to long-term complications, such as effects of donor liver steatosis, MetS and its associated complications, NAFLD, CVD, and CKD, as well as malignancy on the graft and recipient outcome [3, 9, 10]. Due to high rate of MetS and its individual components in the post-LT setting (mainly due to immunosuppressive medications), liver transplant recipients (LTR) have a high risk of graft steatosis and fibrosis (i.e., de novo or recurrent NAFLD). According to the data, MetS affects one out of every two LTR and accounts for up to 42% of CVD-related mortality [9, 11, 12]. Therefore, early recognition of graft steatosis and fibrosis are key issues to prevent adverse outcomes. Although liver biopsy (LB) is still the gold standard for the detection of steatosis, inflammation, and fibrosis, it is an invasive procedure, and LTR can be reluctant to undergo repetitive protocol biopsies [6]. In general population, noninvasive methods for steatosis and fibrosis detection and staging, such as transient elastography (TE) with a controlled attenuation parameter (CAP), have gained popularity in the last 5–10 years [3, 6]. Recently, study data revealed CAP and liver stiffness measurement (LSM) are the good methods for assessment of steatosis and fibrosis in NAFLD patients [13].

According to our best knowledge, there are only two studies that investigated the use of TE with CAP in the post-LT setting [6, 7]. Therefore, the aim of our study was to investigate the prevalence and risk factors of increased CAP and LSM as surrogate markers of liver steatosis and fibrosis in the Croatian Transplant Center that has one of the highest LT rates in the world.

## 2. Patients and Methods

### 2.1. Patients

In this prospective, cross-sectional study, we have evaluated 175 LTRs with a functioning graft for more than one year who came for regular outpatient examinations to the Department of Gastroenterology, University Hospital (UH) Merkur, Zagreb, Croatia, during the 10-month period between October 2019 and August 2020. All included LTRs were at least 18 years old at the time of TE measurements, while recipients with pregnancy, elevation of aminotransferases >5 times the upper limit of normal, as well as those with cholestasis, those with an excessive alcohol consumption (>20 g per day for men and >10 g per day for women), those with failed TE measurements, and those with missing data were not a part of this analysis. Additionally, recipients with malignancy, ascites, right-side heart failure, and valvular heart disease were excluded as well. The study was performed in accordance with the ethical guidelines of the Helsinki and was approved by the ethics committee of UH Merkur.

### 2.2. Objectives

The primary objective of this study was to evaluate the prevalence of increased CAP and LSM as surrogate markers of liver steatosis and fibrosis in LTR. Secondary objectives were to determine the predictors of increased CAP and LSM in population of LTR.

### 2.3. Clinical and Laboratory Data

#### 2.3.1. Recipients and Donor's Data

After the surgical procedure of LT, all recipients were managed in the intensive care unit (ICU) with a standard triple immunosuppressive regimen (corticosteroids, mycophenolate mofetil, and calcineurin inhibitors) as well as postoperative antibiotic therapy and valganciclovir according to the CMV status.

The following recipients' data were analyzed in this study: age, age at LT, gender, aetiologias of ESLD, type of immunosuppressive regimen, recipient's age at the time of TE measurements, time from LT to TE examination, and presence of MetS components (diabetes mellitus, hypertension, obesity, and dyslipidemia). Donor age and body mass index (BMI) were analyzed as well. Obesity was defined as BMI ≥30 kg/m^2^. Hypertension was defined in LTR with a blood pressure ≥130/80 mm Hg or using antihypertensive medications, while diabetes as fasting glucose ≥7.1 mmol/L or use of at least 1 oral hypoglycemic drug or insulin. Finally, dyslipidemia was defined by positive medical history, using of lipid-lowering drugs, or if the serum total cholesterol level was ≥5.2 mmol/L, serum triglyceride (TG) level ≥1.7 mmol/L, and serum high-density lipoprotein (HDL) cholesterol level ≥3.4 mmol/L. Relevant clinical details were obtained from all patients at the time of TE measurements. Laboratory data (using standard laboratory methods) included complete blood cell count, liver tests (total bilirubin, serum aspartate aminotransferase (AST), alanine aminotransferase (ALT), gamma-glutamyl transferase (GGT), and alkaline phosphatase (ALP)), lipidogram (total cholesterol, LDL-cholesterol, HDL-cholesterol, and triglycerides), glucose, and high sensitivity C-reactive protein (hs-CRP).

### 2.4. Transient Elastography

All patients underwent TE measurements after overnight fasting using FibroScan® 502 Touch (Echosens, Paris, France), which was performed using M or XL probe by a certified investigator. Only cases with 10 successful measurements were included in this study. Examinations with an interquartile range/median ratio >30% were excluded because of unreliable results. CAP was used as a surrogate parameter for graft steatosis and was expressed in dB/m, while LSM was used as a surrogate parameter for graft fibrosis and was expressed in kPa. Measurements were performed in decubital position with right arm placed above the patients' head, in the neutral respiratory position, while suspending breathing. We did not have adverse events related to the use of the FibroScan device.

Accordingly, TE patients were considered to have hepatic steatosis if the controlled attenuation parameter (CAP) was ≥238 dB [14]. Severe steatosis was considered if the CAP was ≥290 dB/m. Moderate liver fibrosis (≥*F*2) was considered as a LSM ≥7 kPa and advanced fibrosis (≥*F*3) if LSM was ≥9.6 kPa using the M probe or ≥9.3 kPa using the XL probe [15, 16].

### 2.5. Statistical Analysis

Categorical variables are shown as percentages and continuous variables as means with standard deviation or medians with interquartile range (25^th^ and 75^th^ percentiles) depending on the distribution. Distribution was assessed using the D'Agostino-Pearson test and graphically. Distribution relationship between categorical variables values was tested using the *χ*^2^-test and if necessary, Fisher's exact test. Difference between two continuous variables was tested using the two-way *t*-test for parametric or Mann–Whitney *U* test for nonparametric analysis. Multivariable logistical regression analyses were conducted to identify patient characteristics independently associated with liver steatosis and fibrosis according to the transient elastography. Univariate analysis was first performed on each variable of the independent variables to select variables for the multivariable analyses. Those factors with a *p* value < 0.5 in the univariate analyses were selected as candidate variables for backward multivariable logistical regressions. All the statistical analyses were performed using SPSS V.22.0 (SPSS Inc., Chicago, Illinois, USA). Statistical tests were two-tailed, and significance was set at 0.05.

## 3. Results

### 3.1. Patient Characteristics

A total of 175 patients were included in this study and underwent FibroScan assessment. The mean age of the total study population was 61 (53–65), and 68% (119/175) of them were male. The average BMI of the study population was 28.4 (23.69–31.99) kg/m^2^, while the prevalence of obesity was 34.28% (60/175). In FibroScan assessment, M probe was used in 121 (69.14%) and XL probe in 54 (30.85%) patients, respectively. Furthermore, 64.00% (112/175) patients had hypertension, 38.28% (67/175) diabetes, and 58.85% (103/175) hyperlipidemia. Overall, 118 patients (67.42%) had the echobright liver on abdominal ultrasound (i.e., liver steatosis based on abdominal ultrasound finding).

### 3.2. Prevalence and Predictors of Liver Steatosis and Severe Liver Steatosis

In our population, the prevalence of liver steatosis was 68.57% (120/55), while the prevalence of severe liver steatosis was 46.85% (82/93). Patient characteristics with and without increased CAP are shown in Table 1, while patient characteristics with and without severe liver steatosis are shown in Table 2.

Patients with increased liver steatosis were more often males and had most often alcoholic liver disease as the pretransplant cause of liver cirrhosis, higher proportion of arterial hypertension, higher levels of blood glucose, GGT, triglycerides, total cholesterol, and LDL, and lower levels of HDL. Also, patients with increased liver steatosis had higher levels of LSM (7.2 vs. 5.8 kPa, *p* = 0.012) and higher time from LT to TE (Table 1). On multivariate analysis, independent factors associated with liver steatosis were male gender and total cholesterol as positive predictor and HDL as negative predictor (Table 3).

Furthermore, patients with severe steatosis were older and had higher BMI (30.44 vs. 26.51 kg/m^2^, *p* = 0.038) and consequently higher proportion of obesity, diabetes, blood glucose, and LSM levels (7.4 vs. 6.7 kPa, *p* = 0.019) (Table 2). On multivariate analysis, independent factors positively associated with severe liver steatosis were higher BMI and higher triglyceride levels (Table 4).

### 3.3. Prevalence and Predictors of Moderate Liver Fibrosis and Advanced Liver Fibrosis

In our population, the prevalence of moderate liver fibrosis was 54.85 (96/81), while the prevalence of advanced liver fibrosis was 24% (42/135). Patients characteristics with and without moderate fibrosis are shown in Table 5, while patient characteristics with and without advanced fibrosis are shown in Table 6.

Patients with moderate liver fibrosis had a higher prevalence of arterial hypertension and higher levels of ALT, AST, GGT, and CAP (298 vs. 267 dB, *p* = 0.004) in addition to longer time from performance of LT to TE. Also, patients with moderate fibrosis were more often treated with cyclosporine and mycophenolate mofetil and less often with tacrolimus (Table 5). On multivariate analysis, independent factors positively associated with moderate fibrosis were GGT and CAP (Table 7).

Furthermore, patients with advanced fibrosis had higher levels of total bilirubin, AST, ALT, GGT, and LDM and longer time from LT to TE. As in moderate fibrosis, patients with advanced fibrosis were also more often treated with cyclosporine and mycophenolate mofetil compared to patients without advanced fibrosis (Table 6). On multivariate analysis, an independent factor positively associated with advanced fibrosis was GGT (Table 8).

## 4. Discussion

To the best of our knowledge, this is the third observational study [6, 7] aimed to investigate graft injury noninvasively with CAP and LSM obtained by FibroScan as a surrogate marker of steatosis and fibrosis, which reveals a high prevalence of post-LT steatosis that was associated with the MetS and liver graft fibrosis. NAFLD affects about 25% of the total population, and it is closely related to diabetes mellitus, hypertension, dyslipidemia, and obesity, i.e., the MetS components. Today, we know that NAFLD is the liver manifestation of MetS. Metabolic syndrome and its individual components often develop in the post-LT setting, and immunosuppressive therapy is the main trigger that promotes individual MetS components development [5–7, 9]. According to our results, the prevalence of diabetes, hypertension, obesity, and dyslipidemia was 38.3%, 64%, 34.3%, and 58.85%, respectively. Steatosis after LT has attracted increasing research interest during the last decade. Few authors have published their retrospective studies in which steatosis was defined by LB [17–19]. In our cohort of LTRs, the prevalence of increased CAP values (i.e., steatosis) was 68.57%, while the prevalence of severe liver steatosis was 46.85%. As it was mentioned, there are only two more studies to data that investigated the usefulness of TE with CAP for steatosis and fibrosis detection in the post-LT setting. In the study by Karlas et al. [6], the prevalence of steatosis was 44%, while the prevalence of advanced steatosis was 24%, which is lower than in our study. This can be explained by the fact that in the study by Karlas et al. [6], CAP was not available using the XL probe, and thus, only the M probe was used [6]. Our results closely resembled the results from Chayanupatkul et al. [7], in which TE with CAP was also used as a method for post-LT NAFLD. They reported that 70% of their LTRs had liver steatosis noted on TE; 7.3% LTRs had mild steatosis, 34.7% had moderate steatosis, and 28.0% of LTRs had severe steatosis. According to the literature, the prevalence of steatosis varies across studies, which is probably the consequence of different criteria and methods that were used for steatosis definition. For example, Dumortier et al. [18] analyzed patients who were mainly transplanted for alcoholic liver disease and found that a histological diagnosis of steatosis was present in 131 (31.1%) of the remaining 421 LTRs. Similarly, another biopsy-based study reported the prevalence of allograft steatosis of 40% [19]. Of this, 58% LTRs had mild steatosis while 42% had moderate steatosis [19]. A study from Mayo Clinic reported the prevalence of steatosis of 48% after 10 years post-LT [20]. In our study, the prevalence of steatosis was higher than the aforementioned studies but quite similar to results by Chayanupatkul et al. [7], which also used TE as a method for steatosis assessment in the post-LT setting. However, according to the largest study with protocol biopsy in the context of post-LT NAFLD, NAFLD was present in 67% of the patients with de novo NAFLD and in 100% of the patients with recurrent NAFLD one year after LT [20].

In line with the study by Chayanupatkul et al. [7], in our study, male gender, older age, and alcoholic liver disease as an indication for LT were the risk factors for post-LT steatosis. Contrary to our results, a recent study reported that younger age at time of LT was a risk factor for post-LT steatosis [21], which may be explained by the type of LT [5–7]. Namely, in our study, we have had deceased donors, while Miyaaki [22] et al. had living donors. Recently, we have shown that CAP values were strongly associated with all components of MetS [23–25] in the pre-LT setting. In line with our previous results [23–25], this study confirms that CAP as a surrogate marker of steatosis is related to MetS components also in the post-LT setting. Namely, in our study, CAP was associated with hypertension, higher levels of glucose in blood, and dyslipidemia. Moreover, LTRs with severe steatosis (CAP ≥ 290 db/m) were more obese and had a higher prevalence of diabetes, while independent predictors of severe steatosis were obesity and dyslipidemia. Other biopsy-proven studies confirmed that steatosis post-LT is related to MetS and its individual components, and a study that used CAP, as well as our study, for steatosis detections, also reported that CAP is related to MetS components [6, 18]. Interestingly, in our study, liver enzymes were not related to higher CAP values (severe steatosis), which is similar to results of other authors [7] and to earlier observation that about 50% of NAFLD patients in the pre-LT setting have normal liver tests [23–25].

In the second part of our analysis, we have investigated the prevalence of increased LSM as a surrogate marker of liver fibrosis. In our population, the prevalence of moderate liver fibrosis was 54.85%, while the prevalence of advanced liver fibrosis was 24%. These results may be a consequence of a higher rate of MetS and its individual components in our cohort of LTRs. In contrast to the study by Chayanupatkul et al. [7], in our study, AST, ALT, and GGT were associated with moderate and advanced fibrosis, while in multivariate analysis, GGT was an independent predictor of moderate and advanced fibrosis. However, we have to keep in mind that various factors can influence the increased level of liver enzymes in LTRs. Second, we still do not know what is the true “normal” range for ALT in this population of patients [7]; thus, we cannot reliably use ALT as a marker for further studies when it comes to steatosis (i.e., NAFLD) screening in the context of LTRs [7]. Hypertension was related to the moderate fibrosis, which is in line with the data from the pre-LT setting where hypertension is a risk factor for fibrosis progression [23–25]. Interestingly, although this association did not persist in multivariate analysis, LTRs with moderate and advanced fibrosis were more often treated with cyclosporine and less often with tacrolimus. In contrast to our result, in a study by Dumortier et al. [18], one-third of their analyzed LTRs had perisinusoidal fibrosis, and 4% of LTRs had NASH. Factors that were related to the post-LT steatosis were MetS and its individual components, tacrolimus-based immunosuppressive therapy, alcoholic liver disease as the primary indication for LT, and liver graft steatosis [18]. Our result may partially explain the fact that, in our transplant center, we keep the tacrolimus concentrations in blood within the lowest possible range; thus, their negative effect on the kidneys, hypertension, and diabetes is minimalized. Furthermore, prospective studies that will investigate the influence of immunosuppressive therapy on CAP and LSM values are needed.

The exact role of post-LT steatosis and effects of pretransplant donor steatosis on it are not completely elucidated yet. According to our results and similar to other two studies [6, 7] that used TE with CAP in the post-LT setting, CAP was associated with increased LSM (i.e., fibrosis). Although, in recent biopsy-based study in the pre-LT setting [13], LSM measurements have not been affected by CAP (steatosis); our results as well as results of other two studies [6, 7] indicate an association of allograft steatosis and fibrosis. Namely, we cannot rule out an impact of inflammation (i.e., steatohepatitis) on our measurements because ongoing graft inflammation could be associated with increased LSM even in cases where significant and advanced fibrosis is not present. Regarding our previous experience with TE with CAP, we strongly believe that elevated LSM in LTRs could be a parameter for previous or ongoing graft damage [6, 23–25]. Similar results were observed by Karlas et al. five years ago [6]. As it was mentioned, the rule of post-LT steatosis (i.e., NAFLD) is not completely investigated and understood. A recent study by Gitto et al. [26] reported that de novo NAFLD was associated with adverse CVD events and extrahepatic malignancy, and biopsy-proven NASH was related to the higher long-term LTRs mortality. Thus, but mainly regarding the results in the pre-LT setting, CAP as a surrogate marker of steatosis could become a growing clinical relevance for the follow-up of LTRs because it is easy to use, and it is a noninvasive method for steatosis detection [5–7, 9]. By using TE with CAP to detect and assess the degree of steatosis in LTRs, we could motivate transplant physicians to aggressively treat MetS and its individual components, obesity, diabetes, hypertension, and dyslipidemia [5–7, 9]. Therefore, further investigations in the post-LT setting should answer on the question whether monitoring the changes in the CAP and LSM could be useful for evaluating the treatment of the MetS and the effect of treatment of MetS and its components on de novo and recurrent NAFLD [5–7, 9]. Post-LT steatosis (i.e., NAFLD) is not only important for liver-related mortality but also for some extrahepatic diseases [9]. Namely, today, we know from the data from the pre-LT setting that NAFLD is a multisystem disease that is a risk factor for CVD, CKD, and diabetes type 2, as well as a risk factor for some malignancies such as colorectal cancer [1]. On the other hand, the high incidence of long-term complications after LT such as CKD and CVD suggests the need for a stratification model to identify LTRs at a high risk of developing CKD and CVD post-LT [5–7, 9]. Consequently, further investigations should answer on the question will early NAFLD recognition in the post-LT setting help us to identify those LTRs that are at high risk of CKD and CVD development. In this context, CAP as a surrogate marker of steatosis could have a role [9] because CAP, as a surrogate marker of NAFLD in the pre-LT setting, showed a correlation with cardiovascular risk and CKD [9, 27–30]. Considering this association, the question is whether patients with increased CAP and specifically an increased LSM could benefit from much earlier and much stronger screening for CVD and CKD [9]. We are questioning whether CAP and LSM could be a surrogate marker of subclinical atherosclerosis and consequent markers of increased CVD risk in the post-LT setting [9]. Further studies on this topic are needed. Earlier studies addressed the limitations of the M probe in patients with higher BMI, which led to the development of the XL probe that is specially designed for obese people [5, 27].

Additionally, earlier data addressed that graft fibrosis may in occur in high proportion of LTRs who have normal transaminase levels [5–7]. On the other hand, in our center as well as in many other transplant centers, protocol biopsies are not a part of standard care of LTRs. Consequently, LSM could be a good noninvasive method for the selection of those LTRs that are at risk and who need LB [5–7]. However, the optimal LSM cutoff for detecting each stage of liver fibrosis in the post‐LT setting has not been defined yet and need further studies [5]. Finally, by TE with CAP as a noninvasive method, we could routinely monitor steatosis and fibrosis progression in LTRs in everyday clinical practice [9].

Our study has the strength of the use of one of the more investigated noninvasive imaging methods for measuring liver steatosis and fibrosis. In addition, CAP measurement was assessed by using both FibroScan probes (M and XL). However, our study has few limitations. For the first, cross-sectional design of this study precludes any causal inferences about the directionality of the connections investigated in our study, its dynamics in time, and effects of graft and recipient outcomes. Second, we have used TE with CAP, instead of LB. This makes it impossible to evaluate the initial finding of steatosis in the graft and its dynamics on the posttransplantation finding. However, LB is an invasive procedure, and in our transplant center, we do not perform protocol biopsies. Instead of LB, we have used TE with CAP that is the best investigated and validated noninvasive imaging elastographic method for steatosis and fibrosis detection and quantification. Finally, CAP and LSM are not investigated in the post-LT setting, and we do not know the optimal cutoff values of CAP and LSM for each steatosis and fibrosis stage. However, in our population of LTRs, we have shown that metabolic risk factors (i.e., Mets and its individual components) are associated with CAP measurements, as well as they are associated in the pretransplant setting.

In conclusion, in our study, the prevalence of increased CAP values (i.e., steatosis) was 68.57%, while the prevalence of severe liver steatosis was 46.85%. Moreover, more than half of our LTRs had moderate elevation of LSM (i.e., fibrosis), while the prevalence of advanced liver fibrosis was 24%. Metabolic syndrome components were highly present in our cohort of patients (as well as in other studies) and were associated with CAP and LSM values as well as in the pretransplant setting. Regarding the earlier observations and our result about the high prevalence of metabolic comorbidities and NAFLD after LT and the lack of the abnormal liver test in a significant number of these patients, we strongly believe that TE with CAP may be a reasonable initial assessment for LTRs patients with one or more components of the MetS. As LTRs are living longer post-LT, it is important to investigate the long-term impact of NAFLD on survival of this population of patients [5–7]. Also, it is important to investigate the relationship of NAFLD with CVD and CKD morbidity and mortality in the post-LT setting. In the future, the investigations with protocol biopsies will have to analyze whether CAP and LSM as a surrogate marker of steatosis and fibrosis can be used in prediction of clinically relevant end points (liver related and nonliver related) in LTRs.

## Figures and Tables

**Table 1:** Comparison of groups with and without steatosis (elevated CAP ≥238 dB).

Variables	Steatosis CAP ≥238 dB (*n* = 120)	No steatosis CAP <238 dB (*n* = 55)	*p* value
Age at LT, years (IQR)	55 (50–61)	57 (43–60)	0.286
Age at TE, years (IQR)	61 (54–65)	60 (46–65)	0.131
Donor age, years	60 (48–70)	57 (41–68)	0.596
Male, % (*n*)	75.83 (91)	52.72 (29)	0.006^*∗*^

Cause of liver disease, % (*n*)			
Autoimmune liver disease	6.67 (8)	0.2 (11)	0.005^*∗*^
NAFLD	0.83 (1)	0.0 (0)	
Alcoholic liver disease	37.5 (45)	18.18 (10)	
HCV	5.0 (6)	0.0 (0)	
Others	50.0 (60)	61.8 (34)	

BMI, kg/m^2^ (IQR)	28.76 (23–32)	26.67 (24–30)	0.402

BMI category, % (*n*)			
Normal <25	37.5 (45)	36.4 (20)	0.246
Overweight 25–29.9	23.3 (28)	40.0 (22)	
Obese ≥30	39.2 (47)	23.6 (13)	

Donors BMI, kg/m^2^ (IQR)	26.23 (24–28)	26.03 (24–28)	0.911

Donors BMI category, % (*n*)			
Normal <25	40.0 (48)	34.54 (19)	0.627
Overweight 25–29.9	44.2 (53)	52.73 (29)	
Obese ≥30	15.8 (19)	12.73 (7)	

Hypertension, % (*n*)	69.75 (83)	52.73 (29)	0.041^*∗*^
Diabetes, % (*n*)	43.70 (52)	27.27 (15)	0.063
Thrombocytes x10^9^/L	167 (135–226)	170 (136–217)	0.632
Glucose, mmol/L (IQR)	6.4 (6–8)	5.8 (5–6)	<0.001^*∗*^
Total bilirubin, mg/dL (IQR)	16 (12–21)	15 (12–26)	0.558
ALT, U/L (IQR)	29 (20–39)	26 (17–34)	0.186
AST, U/L (IQR)	28 (23–40)	28 (22–37)	0.598
GGT, U/L (IQR)	41 (24–94)	31 (17–84)	0.041^*∗*^
Triglyceride, mmol/L (IQR)	1.37 (1.1–2.0)	1.03 (0.8–1.4)	<0.001^*∗*^
Total cholesterol, mmol/L (IQR)	5.1 (4.5–5.9)	4.7 (4.1–5.5)	0.025^*∗*^
LDL, mmol/L (IQR)	3.1 (2.6–3.7)	2.8 (2.1–3.5)	0.016^*∗*^
HDL, mmol/L (IQR)	1.3 (1.1–1.6)	1.4 (1.2–1.7)	0.033^*∗*^
CRP, mg/L (IQR)	3.4 (2–6)	2.3 (1–4)	0.066
LSM, kPa (IQR)	7.2 (6.0–9.0)	5.8 (4.5–9.4)	0.012^*∗*^

Immunosuppression, % (*n*)			
Tacrolimus	67.5 (81)	76.4 (42)	0.311
Cyclosporine	32.5 (39)	21.8 (12)	
Prednisone	3.3 (4)	7.3 (4)	
mTOR inhibitor	(0)	1.8 (1)	
Mycophenolate mofetil	70.0 (84)	65.5 (36)	
Azathioprine	(0)	5.5 (3)	

Time from LT to TE, years (IQR)	4 (3–6)	3 (2–5)	0.033^*∗*^

^*∗*^LT, liver transplantation; NAFLD, nonalcoholic fatty liver disease; HCV, hepatitis C virus infection; BMI, body mass index; AST, serum aspartate aminotransferase; ALT, alanine aminotransferase; GGT, gamma-glutamyl transferase; CRP, C-reactive protein; LSM, liver stiffness measurement; TE, transient elastography; CAP, controlled attenuation parameter.

**Table 2:** Comparison of groups with and without severe steatosis (elevated CAP ≥290 dB).

Variables	Severe steatosis CAP ≥290 dB (*n* = 82)	No severe steatosis CAP <290 dB (*n* = 93)	*p* value
Age at LT, years (IQR)	55 (50–61)	55 (45–61)	0.412
Age at TE, years (IQR)	64 (55–69)	60 (51–65)	0.039^*∗*^
Donor age, years	57 (42–68)	59 (45–67)	0.711
Male, % (*n*)	71.95 (59)	64.52 (60)	0.374

Cause of liver disease, % (*n*)			
Autoimmune liver disease	8.54 (7)	12.90 (12)	0.211
NAFLD	1.22 (1)	(0)	
Alcoholic liver disease	39.02 (32)	24.73 (23)	
HCV	3.66 (3)	3.23 (3)	
Others	47.56 (39)	59.14 (55)	

BMI, kg/m^2^ (IQR)	30.44 (26–34)	26.51 (23–30)	0.038^*∗*^

BMI category, % (*n*)			
Normal <25	26.8 (22)	43.0 (40)	0.045^*∗*^
Overweight 25–29.9	26.8 (22)	31.2 (29)	
Obese ≥30	46.4 (38)	25.8 (24)	

Donors BMI, kg/m^2^ (IQR)	26.57 (24–29)	26.23 (24–28)	0.877

Donors BMI category, % (*n*)			
Normal <25	40.2 (33)	35.5 (33)	0.266
Overweight 25–29.9	41.5 (34)	52.7 (49)	
Obese ≥30	18.3 (15)	11.8 (11)	

Hypertension, % (*n*)	71.95 (59)	58.06 (54)	0.079
Diabetes, % (*n*)	50.00 (41)	27.96 (26)	0.005^*∗*^
Thrombocytes x10^9^/L	166 (134–218)	170 (136–228)	0.652
Glucose, mmol/L (IQR)	6.4 (6–8)	6 (5–7)	0.018^*∗*^
Total bilirubin, mmol/L (IQR)	16.5 (12–22)	14.0 (12.22)	0.732
ALT, U/L (IQR)	29.5 (20–39)	26.0 (18–36)	0.056
AST, U/L (IQR)	27.5 (22–43)	28 (23–39)	0.319
GGT, U/L (IQR)	42 (24–99)	36 (21–80)	0.191
Triglyceride, mmol/L (IQR)	1.4 (1.1–2.1)	1.1 (0.9–1.5)	0.004
Total cholesterol, mmol/L (IQR)	5.2 (4.5–6)	4.9 (4.4–5.6)	0.168
LDL, mmol/L (IQR)	3.1 (2.5–3.7)	3 (2.4–3.5)	0.340
HDL, mmol/L (IQR)	1.26 (1.0–1.6)	1.34 (1.1–1.6)	0.227
CRP, mg/L (IQR)	3.7 (2–6)	2.5 (1–5)	0.755
LSM, kPa (IQR)	7.4 (6.3–9.2)	6.7 (5.0–9.0)	0.019^*∗*^

Immunosuppression, % (*n*)			
Tacrolimus	68.3 (56)	72.0 (67)	0.356
Cyclosporine	31.7 (26)	28.0 (26)	
Prednisone	1.2 (1)	8.0 (7)	
mTOR inhibitor	(0)	1.1 (1)	
Mycophenolate mofetil	65.9 (54)	72.0 (67)	
Azathioprine	(0)	3.2 (3)	

Time from LT to TE, years (IQR)	5 (3–6)	3 (2–5)	0.099

^*∗*^LT, liver transplantation; NAFLD, nonalcoholic fatty liver disease; HCV, hepatitis C virus infection; BMI, body mass index; AST, serum aspartate aminotransferase; ALT, alanine aminotransferase; GGT, gamma-glutamyl transferase; CRP, C-reactive protein; LSM, liver stiffness measurement; TE, transient elastography.

**Table 3:** Univariate and multivariate analysis of predictors of steatosis (elevated CAP ≥238 dB).

Variables	Univariate		Multivariate	
	OR (95% CI)	*p* value	OR (95% CI)	*p* value
Age at LT, years	1.04 (1.01–1.07)	0.026^*∗*^	0.94 (0.91–1.09)	0.445
Age at TE, years	1.04 (1.01–1.07)	0.008^*∗*^	1.05 (0.90–1.22)	0.494
Donor age, years	0.99 (0.97–1.01)	0.462		
Gender (female ref.)	2.78 (1.42–5.46)	0.003^*∗*^	2.73 (1.25–5.94)	0.011^*∗*^

Cause of liver disease				
Autoimmune liver disease	0.29 (0.11–0.76)	0.012^*∗*^	0.50 (0.16–1.57)	0.239
Alcoholic liver disease	2.74 (1.26–5.96)	0.011^*∗*^	1.61 (0.67–4.03)	0.277
Others	0.61 (0.32–1.17)	0.134		
BMI, kg/m^2^	1.03 (0.94–1.13)	0.509		
Donors BMI, kg/m^2^	1.01 (0.93–1.10)	0.867		
Hypertension	2.08 (1.07–3.99)	0.031^*∗*^	1.23 (0.52–2.90)	0.631
Diabetes	2.67 (1.03–4.15)	0.040^*∗*^	0.85 (0.32–2.23)	0.749
Thrombocytes x10^9^/L	1.01 (0.99–1.02)	0.545		
Glucose, mmol/L	1.37 (1.06–1.78)	0.017^*∗*^	1.07 (0.81–1.42)	0.605
Total bilirubin, mmol/L	1.98 (0.95–1.01)	0.206		
ALT, U/L	1.01 (0.99–1.02)	0.592		
AST, U/L	1.01 (0.99–1.02)	0.385		
GGT, U/L	1.01 (0.99–1.02)	0.332		
Triglyceride, mmol/L	2.67 (1.46–4.90)	0.002^*∗*^	1.19 (0.16–8.54)	0.862
Total cholesterol, mmol/L	1.47 (1.07–2.02)	0.018^*∗*^	7.46 (1.78–31.15)	0.006^*∗*^
LDL, mmol/L	1.61 (1.10–2.35)	0.015^*∗*^	1.21 (0.91–1.46)	0.065
HDL, mmol/L	0.39 (0.17–0.91)	0.023^*∗*^	0.09 (0.02–0.42)	0.002^*∗*^
CRP, mg/L	0.99 (0.96–1.01)	0.305		
LSM, kPa	1.03 (0.97–1.09)	0.287		
Time from LT to TE, years	1.14 (0.99–1.30)	0.065		

^*∗*^LT, liver transplantation; NAFLD, nonalcoholic fatty liver disease; HCV, hepatitis C virus infection; BMI, body mass index; AST, serum aspartate aminotransferase; ALT, alanine aminotransferase; GGT, gamma-glutamyl transferase; CRP, C-reactive protein; LSM, liver stiffness measurement; TE, transient elastography.

**Table 4:** Univariate and multivariate analysis of predictors of severe steatosis (elevated CAP ≥290 dB).

Variables	Univariate		Multivariate	
	OR (95% CI)	*p* value	OR (95% CI)	*p* value
Age at LT, years	1.02 (0.99–1.05)	0.115		
Age at TE, years	1.03 (1.01–1.06)	0.049^*∗*^	1.01 (0.96–1.07)	0.494
Donor age, years	0.99 (0.97–1.02)	0.574		
Gender (female ref.)	1.48 (0.77–2.82)	0.240		

Cause of liver disease				
Autoimmune liver disease	0.64 (0.24–1.71)	0.372	1.52 (0.91–1.83)	0.984
Alcoholic liver disease	1.99 (1.04–3.80)	0.038^*∗*^		
HCV	1.15 (0.23–5.88)	0.863		
Others	0.61 (0.33–1.11)	0.108		
BMI, kg/m^2^	1.10 (1.01–1.20)	0.042^*∗*^	2.77 (1.34–3.85)	0.048^*∗*^
Donors BMI, kg/m^2^	1.01 (0.93–1.09)	0.836		
Hypertension	0.10 (0.01–1.54)	0.100		
Diabetes	2.64 (1.41–4.95)	0.002^*∗*^	1.48 (1.21–1.85)	0.044^*∗*^
Thrombocytes x10^9^/L	0.99 (0.98–1.01)	0.621		
Glucose, mmol/L	1.21 (1.02–1.45)	0.033^*∗*^	1.11 (0.95–1.30)	0.368
Total bilirubin, mmol/L	1.01 (0.98–1.04)	0.718		
ALT, U/L	1.01 (0.99–1.02)	0.534		
AST, U/L	1.01 (0.99–1.02)	0.309		
GGT, U/L	1.01 (0.98–1.02)	0.184		
Triglyceride, mmol/L	1.79 (1.18–2.69)	0.006^*∗*^	1.63 (1.15–2.59)	0.041^*∗*^
Total cholesterol, mmol/L	1.23 (0.93–1.63)	0.146		
LDL, mmol/L	1.19 (0.86–1.66)	0.290		
HDL, mmol/L	0.60 (0.27–1.34)	0.209		
CRP, mg/L	0.99 (0.97–1.02)	0.770		
LSM, kPa	1.02 (0.97–1.07)	0.410		
Time from LT to TE, years	1.09 (0.98–1.23)	0.104		

^*∗*^LT, liver transplantation; NAFLD, nonalcoholic fatty liver disease; HCV, hepatitis C virus infection; BMI, body mass index; AST, serum aspartate aminotransferase; ALT, alanine aminotransferase; GGT, gamma-glutamyl transferase; CRP, C-reactive protein; LSM, liver stiffness measurement; TE, transient elastography.

**Table 5:** Comparison of groups with and without moderate fibrosis.

Variables	Moderate fibrosis, *N* = 96	No moderate fibrosis, *N* = 81	*p* value
Age at LT, years (IQR)	55 (46–60)	56 (49–62)	0.288
Age at TE, years (IQR)	61 (53–64)	60 (53–66)	0.818
Donor age, years	57 (44–68)	60 (44–67)	0.971
Male, % (*n*)	68.1 (64)	68.7 (55)	0.945

Cause of liver disease, % (*n*)			
Autoimmune liver disease	8.3 (8)	14.8 (12)	0.280
NAFLD	0	1.2 (1)	
Alcoholic liver disease	36.5 (35)	27.2 (22)	
HCV	2.1 (2)	4.9 (4)	
Others	53.1 (51)	51.9 (42)	

BMI, kg/m^2^ (IQR)	28.4 (24–32)	27.5 (23–32)	0.870

BMI category, % (*n*)			
Normal <25	35.4 (34)	38.3 (31)	0.737
Overweight 25–29.9	32.3 (31)	23.4 (19)	
Obese ≥30	32.3 (31)	38.3 (31)	

Donors BMI, kg/m^2^ (IQR)	26.3 (25–29)	26 (23–28)	0.132

Donors BMI category, % (n)			
Normal <25	31.2 (30)	44.4 (36)	0.105
Overweight 25–29.9	55.2 (53)	39.5 (32)	
Obese ≥30	13.5 (13)	16.0 (13)	

Hypertension, % (*n*)	72.9 (70)	55.6 (45)	0.024^*∗*^
Diabetes, % (*n*)	41.7 (40)	35.8 (29)	0.521
Thrombocytes x10^9^/L	161 (129–221)	170 (139–219)	0.540
Glucose, mmol/L (IQR)	6.3 (5–8)	6 (5.4–6.9)	0.152
Total bilirubin, mg/dL (IQR)	17 (12–24)	15 (11–19)	0.173
ALT, U/L (IQR)	31 (23–49)	24 (17–33)	<0.001^*∗*^
AST, U/L (IQR)	31 (23–48)	26 (21–34)	0.001^*∗*^
GGT, U/L (IQR)	53 (28–127)	29 (17–43)	<0.001^*∗*^
Triglyceride, mmol/L (IQR)	1.32 (1–1.9)	1.16 (0.9–1.7)	0.095
Total cholesterol, mmol/L (IQR)	5.1 (4.4–5.7)	5.2 (4.5–6.1)	0.572
LDL, mmol/L (IQR)	3 (2.4–3.5)	3.2 (2.5–3.8)	0.133
HDL, mmol/L (IQR)	1.3 (1.1–1.6)	1.3 (1.1–1.6)	0.692
CRP, mg/L (IQR)	3.3 (2–6)	3.1 (1.9–5.8)	0.892
CAP, dB (IQR)	298 (250–334)	267 (203–310)	0.004^*∗*^

Immunosuppression, % (*n*)			
Tacrolimus	62.5 (60)	80.2 (65)	0.016^*∗*^
Cyclosporine	37.2 (35)	21.5 (17)	
Prednisone	5.2 (5)	3.7 (3)	
mTOR inhibitor	1.0 (1)	(0)	
Mycophenolate mofetil	74.0 (71)	61.7 (50)	
Azathioprine	1.0 (1)	2.5 (2)	

Time from LT to TE, years (IQR)	5 (3–6)	3 (2–5)	0.008^*∗*^

^*∗*^LT, liver transplantation; NAFLD, nonalcoholic fatty liver disease; HCV, hepatitis C virus infection; BMI, body mass index; AST, serum aspartate aminotransferase; ALT, alanine aminotransferase; GGT, gamma-glutamyl transferase; CRP, C-reactive protein; CAP, controlled attenuation parameter; TE, transient elastography.

**Table 6:** Comparison of groups with and without advanced fibrosis.

Variables	Advanced fibrosis, *N* = 42	No advanced fibrosis, *N* = 135	*p* value
Age at LT, years (IQR)	53 (44–60)	56 (50–61)	0.061
Age at TE, years (IQR)	58 (51–64)	61 (54–65)	0.159
Donor age, years	59 (46–70)	57 (43–67)	0.806
Male, % (*n*)	71.4 (30)	67.4 (89)	0.768

Cause of liver disease, % (*n*)			
Autoimmune liver disease	7.1 (3)	12.6 (17)	0.578
NAFLD	0	0.7 (1)	
Alcoholic liver disease	40.5 (17)	29.6 (40)	
HCV	4.8 (2)	3.0 (4)	
Others	47.6 (20)	54.1 (73)	

BMI, kg/m^2^ (IQR)	29.35 (23–32)	27 (24–32)	0.738

BMI category, % (*n*)			
Normal <25	40.5 (17)	34.8 (47)	0.487
Overweight 25–29.9	19.0 (8)	32.6 (44)	
Obese ≥30	40.5 (17)	32.6 (44)	
Donors BMI, kg/m^2^ (IQR)	26.54 (25–29)	26 (24–28)	0.312

Donors BMI category, % (*n*)			
Normal <25	31.0 (13)	39.3 (53)	0.620
Overweight 25–29.9	52.4 (22)	46.7 (63)	
Obese ≥30	16.7 (7)	14.1 (19)	

Hypertension, % (*n*)	76.2 (32)	61.5 (83)	0.119
Diabetes, % (*n*)	45.2 (19)	37.0 (50)	0.441
Thrombocytes x10^9^/L	145 (106–221)	170 (144–221)	0.082
Glucose, mmol/L (IQR)	6.2 (6–8)	6.1 (5–7)	0.240
Total bilirubin, mg/dL (IQR)	18 (14–26)	15 (12–20)	0.005^*∗*^
ALT, U/L (IQR)	33 (24–60)	26 (18–35)	<0.001^*∗*^
AST, U/L (IQR)	43 (25–72)	26 (21–35)	<0.001^*∗*^
GGT, U/L (IQR)	83 (40–173)	31 (21–65)	<0.001^*∗*^
Triglyceride, mmol/L (IQR)	1.43 (1.1–1.9)	1.2 (0.9–1.8)	0.066
Total cholesterol, mmol/L (IQR)	5.1 (4.3–5.5)	5.1 (4.5–6)	0.282
LDL, mmol/L (IQR)	3.1 (2.6–3.7)	2.9 (2.3–3.4)	0.048^*∗*^
HDL, mmol/L (IQR)	1.2 (1–1.7)	1.3 (1.1–1.6)	0.429
CRP, mg/L (IQR)	4.4 (2–7)	3.1 (1.8–5.6)	0.368
CAP, dB (IQR)	285 (213–341)	277 (222–315)	0.469

Immunosuppression, % (*n*)			
Tacrolimus	50.0 (21)	77.0 (104)	<0.001^*∗*^
Cyclosporine	52.5 (21)	23.3 (31)	
Prednisone	2.4 (1)	5.2 (7)	
mTOR inhibitor	2.4 (1)	0	
Mycophenolate mofetil	78.6 (33)	65.2 (88)	
Azathioprine	2.4 (1)	1.5 (2)	

Time from LT to TE, years (IQR)	5 (3–6)	4 (2–6)	0.015^*∗*^

^*∗*^LT, liver transplantation; NAFLD, nonalcoholic fatty liver disease; HCV, hepatitis C virus infection; BMI, body mass index; AST, serum aspartate aminotransferase; ALT, alanine aminotransferase; GGT, gamma-glutamyl transferase; CRP, C-reactive protein; CAP, controlled attenuation parameter; TE, transient elastography.

**Table 7:** Univariate and multivariate analysis of predictors of moderate fibrosis.

Variables	Univariate		Multivariate	
	OR (95% CI)	*p* value	OR (95% CI)	*p* value
Age at LT, years	0.99 (0.96–1.02)	0.521		
Age at TE, years	1.00 (0.97–1.03)	0.972		
Donor age, years	1.01 (0.98–1.02)	0.535		
Gender (female ref.)	0.96 (0.51–1.84)	0.925		

Cause of liver disease, % (*n*)				
Autoimmune liver disease	0.52 (0.20–1.35)	0.180		
Alcoholic liver disease	1.54 (0.81–2.92)	0.188		
HCV	0.41 (0.07–2.30)	0.310		
Others	1.05 (0.58–1.90)	0.866		
BMI, kg/m^2^	1.02 (0.93–1.11)	0.692		
Donors BMI, kg/m^2^	1.05 (0.97–1.14)	0.212		
Hypertension, % (*n*)	2.15 (1.15–4.04)	0.017		
Diabetes, % (*n*)	1.28 (0.70–2.36)	0.426		
Thrombocytes x10^9^/L	0.99 (0.98–1.01)	0.844		
Glucose, mmol/L	1.11 (0.96–1.28)	0.171		
Total bilirubin, mmol/L	1.01 (0.98–1.04)	0.433		
ALT, U/L	1.02 (1.01–1.03)	0.019^*∗*^	1.01 (0.97–1.03)	0.717
AST, U/L	1.02 (1.01–1.04)	0.013^*∗*^	1.01 (0.98–1.02)	0.937
GGT, U/L	1.02 (1.01–1.03)	0.001^*∗*^	1.03 (1.01–1.05)	0.001^*∗*^
Triglyceride, mmol/L	1.34 (0.92–1.94)	0.126		
Total cholesterol, mmol/L	0.91 (0.69–1.20)	0.495		
LDL, mmol/L	0.77 (0.55–1.07)	0.125		
HDL, mmol/L	1.15 (0.52–2.54)	0.726		
CRP, mg/L	0.98 (0.94–1.02)	0.325		
CAP, db/m	1.02 (1.01–1.03)	0.003^*∗*^	1.08 (1.02–1.15)	0.010^*∗*^
Time from LT to TE, years	1.15 (1.02–1.30)	0.024^*∗*^	1.06 (0.92–1.22)	0.403

^*∗*^LT, liver transplantation; NAFLD, nonalcoholic fatty liver disease; HCV, hepatitis C virus infection; BMI, body mass index; AST, serum aspartate aminotransferase; ALT, alanine aminotransferase; GGT, gamma-glutamyl transferase; CRP, C-reactive protein; CAP, controlled attenuation parameter; TE, transient elastography.

**Table 8:** Univariate and multivariate analysis of predictors of advanced fibrosis.

Variables	Univariate		Multivariate	
	OR (95% CI)	*p* value	OR (95% CI)	*p* value
Age at LT, years	0.98 (0.95–1.01)	0.146		
Age at TE, years	0.98 (0.95–1.02)	0.309		
Donor age, years	1.01 (0.98–1.02)	0.580		
Gender (female ref.)	1.21 (0.56–2.59)	0.627		

Cause of liver disease, % (*n*)				
Autoimmune liver disease	0.53 (0.15–1.92)	0.337		
Alcoholic liver disease	1.62 (0.79–3.31)	0.191		
HCV	1.64 (0.29–9.27)	0.577		
Others	0.77 (0.39–1.55)	0.465		
BMI, kg/m^2^ (IQR)	1.03 (0.93–1.14)	0.541		
Donors BMI, kg/m^2^ (IQR)	1.05 (0.95–1.15)	0.342		
Hypertension, % (*n*)	2.00 (0.91–4.42)	0.085		
Diabetes, % (*n*)	1.40 (0.70–2.83)	0.342		
Thrombocytes	0.99 (0.98–1.01)	0.468		
Glucose, mmol/L (IQR)	1.13 (0.99–1.28)	0.067		
Total bilirubin, mg/dL (IQR)	1.03 (1.01–1.07)	0.046^*∗*^	1.01 (0.95–1.06)	0.962
ALT, U/L (IQR)	1.02 (1.01–1.03)	0.002^*∗*^	0.98 (0.96–1.01)	0.360
AST, U/L (IQR)	1.04 (1.02–1.05)	<0.001^*∗*^	1.03 (0.99–1.07)	0.064
GGT, U/L (IQR)	1.02 (1.01–1.04)	<0.001^*∗*^	1.04 (1.01–1.06)	0.031^*∗*^
Triglyceride, mmol/L (IQR)	1.46 (1.02–2.11)	0.047^*∗*^		
Total cholesterol, mmol/L (IQR)	0.78 (0.56–1.06)	0.150		
LDL, mmol/L (IQR)	1.35 (1.02–.1.65)	0.040^*∗*^	1.30 (0.90–1.64)	0.126
HDL, mmol/L (IQR)	0.91 (0.36–2.30)	0.837		
CRP, mg/L (IQR)	0.99 (0.95–1.03)	0.640		
CAP, db/m	1.00 (0.99–1.02)	0.491		
Time from LT to TE, years (IQR)	1.13 (0.99–1.27)	0.053		

^*∗*^LT, liver transplantation; NAFLD, nonalcoholic fatty liver disease; HCV, hepatitis C virus infection; BMI, body mass index; AST, serum aspartate aminotransferase; ALT, alanine aminotransferase; GGT, gamma-glutamyl transferase; CRP, C-reactive protein; CAP, controlled attenuation parameter; TE, transient elastography.

## Data Availability

The data used to support the findings of this study are not available due to ethical restrictions.
